# New X-chromosomal interactors of dFMRP regulate axonal and synaptic morphology of brain neurons in *Drosophila melanogaster*

**DOI:** 10.1080/13102818.2014.937897

**Published:** 2014-10-23

**Authors:** Dimitrina Georgieva, Roumen Dimitrov, Meglena Kitanova, Ginka Genova

**Affiliations:** ^a^ Faculty of Biology, Sofia University ‘St. Kliment Ohridski’, Sofia, Bulgaria; ^b^ Institute of Biology and Immunology of Reproduction, Bulgarian Academy of Sciences, Sofia, Bulgaria

**Keywords:** *dfmr*1, *peb/hnt*, *rok*, *sgg*, *ras*, brain axon, synaptic morphology

## Abstract

Fragile X syndrome is a neuro-developmental disease caused by transcriptional inactivation of the gene FMR1 (fragile X mental retardation 1) and loss of its protein product FMRP. FMRP has multiple neuronal functions which are implemented together with other proteins. To better understand these functions, the aim of this study was to reveal new protein interactors of dFMRP. In a forward genetic screen, we isolated ethyl-metanesulphonate-induced X-chromosomal modifier mutations of *dfmr*1. Four of them were identified and belong to the genes: *peb/hindsight*, *rok*, *shaggy* and *ras*. They are dominant suppressors of the *dfmr*1 overexpression wing phenotype ‘notched wings’. These mutations dominantly affected the axonal and synaptic morphology of the lateral ventral neurons (LNv's) in adult *Drosophila* brains. Heterozygotes for each of them displayed effects in the axonal growth, pathfinding, branching and in the synapse formation of these neurons. Double heterozygotes for both *dfmr*1-null mutation and for each of the suppressor mutations showed robust genetic interactions in the fly central nervous system. The mutations displayed severe defects in the axonal growth and synapse formation of the LNv's in adult brains. Our biochemical studies showed that neither of the proteins – Rok, Shaggy, Peb/Hnt or Ras – encoded by the four mutated genes regulates the protein level of dFMRP, but dFMRP negatively regulates the protein expression level of Rok in the brain. Altogether, these data suggest that Rok, Shaggy, Peb/Hnt and Ras are functional partners of dFMRP, which are required for correct wing development and for neuronal connectivity in *Drosophila* brain.

## Introduction

Fragile X syndrome (FraX) is a heritable neurological disorder caused by an abnormal increase of the CGG-triplet number in the promoter region of the gene FMR1 (fragile X mental retardation 1) in humans. This triplet expansion leads to hypermethylation of the CpG-islands and to transcriptional inactivation of FMR1.[1,2]

Patients with FraX, lacking fragile X mental retardation protein 1 (FMRP1 or FMRP), suffer from neuro-developmental brain dysfunction manifested clinically with a series of features, including mental retardation, sleep disturbances and behavioral abnormalities. Unlike most X-linked diseases, *de novo* mutations are rare in patients with FraX syndrome, with the exception of microdeletions and microduplications, which are relatively rare and occur exclusively *de novo*.[3,4]

FMRP is an RNA-binding protein which is most highly expressed in the nervous system.[5] It contains two K homology (KH) domains and one RGG (Arg–Gly–Gly) domain.[6,7] FMRP mainly binds specific mRNAs with intramolecular G-quadruplex structures by means of its RGG box.[8–12] Because of the presence of a nuclear localization signal (NLS) and a nuclear export signal (NES), it is proposed to shuttle between the nucleus and the cytoplasm, to form messenger ribonucleoprotein (mRNP) complexes and to export specific transcripts to the cytoplasm, where the FMRP modulates their localization and protein synthesis.[13–21] FMRP is also involved in the regulation of mRNA stability.[22–24]

FraX has been modelled in mice [25] and *Drosophila*.[26,27] Both animal models recapitulate the main clinical features of the disease: anomalies in dendritic, axonal and synaptic morphology, aberrant development and physiology, altered synaptic plasticity, behavioral disturbances and cognitive impairment (reviewed in [28–30]).

To better understand the functions of FMRP in the nervous system, efforts have been made by different research groups to determine its protein interactors. Ceman et al. [31] used co-immunoprecipitation in a mouse cell culture to identify six proteins within the FMRP-associated mRNP complexes. These included two well-known fragile X-related proteins, FXR1P and FXR2P, and the RNA-binding protein nucleolin. By means of the yeast two-hybrid assay and the N-terminus of FMRP as a bait, additional FMRP interactors, NUFIP (nuclear FMRP interacting protein) and CYFIP1/2 (cytoplasmic FMRP interacting protein 1/2), were reported.[32,33] NUFIP is a nuclear RNA-binding protein which co-localizes with nuclear isoforms of FMRP. It has been hypothesized that FMRP might bind specific mRNAs and form common ribonucleoprotein particles with NUFIP or take part in post-transcriptional processes in the nucleus.[32] CYFIP1/2 was shown to interact with the small GTPase Rac1, implicated in the dynamic reorganization of actin cytoskeleton.[34] These studies associated FMRP to pathways controlling neuronal morphology, connectivity and synaptic plasticity. It has also been shown that *Drosophila* actin-binding protein profilin is negatively regulated by dFMRP.[35] These studies emphasize the role of actin cytoskeleton misregulation in the neuronal features of FraX syndrome.

As mentioned earlier, FMRP has been known to inhibit the translation of neuronal mRNAs. The Tdrd3 protein, which is involved in proteolyti degradation, has been reported to function in the FMRP translational repressor pathway and to interact physically with FMRP.[36] It has been suggested that FMRP inhibits translation trough sequestering mRNAs into translationally silent mRNPs. Under conditions of cellular stress they accumulated in discrete cytoplasmic particles termed stress granules. Formation of such granules containing FMRP was shown to be induced by Tdrd3-overexpression.

In a candidate-based screen for fragile X-dominant interactors in the developing *Drosophila* retina, Cziko et al. [37] uncovered several proteins with a putative function in the dFMRP translational repressor pathway: Dco/Dbt, PABP, Orb2, Rm62 and SmD3. These proteins were found in the dFMRP-positive neuritic RNA granules known to contain translationally repressed mRNAs. The identification of Argonaute (Ago), a key component of the siRNA interference pathway as an interactor of dFMRP, proposed a mechanism to explain how dFMRP regulates the translation of its target mRNAs. It was hypothesized that dFMR1 might exert its function through the multi-protein RISC complex (RNA-induced silencing complex).[38] In a genetic screen of the *Drosophila* autosomes, Zarnescu et al. [39] identified the cytoskeletal protein Lgl as a functional partner of FMRP. They showed that the protein formed a functional complex with FMRP in mice and flies and that this complex was regulated by the polarity complex proteins (PAR) protein complex. Lgl/FMRP/PAR was suggested to function in the sorting of FMRP granules and their transport to the sites of translation. Another report identified several dFMRP interactors which participated in the microtubule transport in the mammalian nervous system. FMRP was found in the same complex with the kinesin heavy chain [40] and direct interactions with the kinesin light chain were also reported.[41] *Drosophila* dFMRP was also shown to interact with motor proteins (kinesin heavy chain and dynein heavy chain).[42] More recently, the microtubule-severing protein Spastin was shown to be an interactor of dFMRP in different processes with a key role in microtubule formation and transport of mitochondria.[43]

Here we present the results of a forward genetic screen in *Drosophila* aimed to find X-chromosomal interactors of dFMRP which function together with it in the wing tissue and in the axonal and synaptic morphology of brain neurons. We isolated mutations in four genes: *peb/hindsight*, *rok*, *shaggy* and *ras*. These mutations dominantly modify the *dfmr*1 overexpression wing phenotype. The protein products, encoded by four genes: the Rho kinase Rok, the Glycogen synthase kinase Shaggy, the transcription factor Peb/Hnt and the inosine monophosphate dehydrogenase (IMPD), affect the axonal and synaptic morphology of brain neurons. For these functions they require dFMRP. A simultaneous decrease of this protein and any of the other interacting proteins cause severe disturbances in axonal growth and synapse formation. Our biochemical studies show that dFMRP negatively regulates the expression level of Rok in the brain. All these data suggest that Rok, Shaggy, Peb/Hnt and Ras are functional partners of dFMRP, which are required for the correct wing development and for the neuronal connectivity in *Drosophila* brain.

## Materials and metods

### *Drosophila* stocks

*Drosophila* flies *w* [1118] were used as wild-type control. We also used the following *Drosophila* stocks for overexpression of the *dfmr*1 gene in the wing imaginal discs: *w*[1118]; *P*{*w*[*+mc*] = *UAS-Fmr.Z*}3 and *w*[*]; *P*{*w*[*+m*] = *GAL*4*-vg.M*}2; *TM*2*/TB*6*B*, *Tb*[1]; or in the adult brain neurons: *w*[*]; *P*{*w*[*+m*] = *GAL*4*-vg.M*}2; *P*{*w*[*+mC*] = *GAL*4*-elav.L*}3*// TB*6*B*, *Tb*[1]. For the deficiency mapping we used a set of stocks from the first chromosome duplication/deficiency kit, obtained from the Bloomington Drosophila Stock Center at Indiana University (USA). For the analysis of the neuron morphology we designed the following stocks:

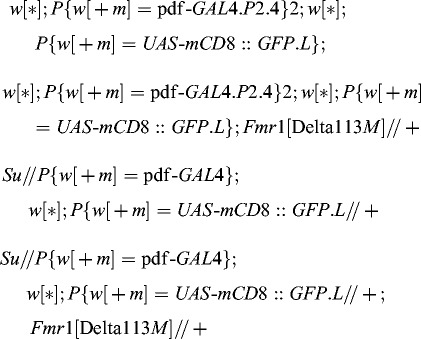



(*Su* is each suppressor mutation from our previous screen: *peb^hnt-E^* ^8^, *rok* ^1^, *sgg^G^* ^0263^ and *ras^G^* ^0380*b*^).[44] *UAS-mCD*8: *GFP.L* is a construct which labels the neuronal membranes and the neuronal projections).

*Fmr*1[Delta113*M*] is a *dfmr*1 null-mutation. It contains a large intragenic deletion.[27] pdf*-GAL*4*.P*2.4 drives the expression of green fluorescence protein (GFP) in the ventral lateral neurons (LNv's) expressing the pigment dispersing factor (PDF), which are the pacemaker neurons in *Drosophila*.[45] Additional information on the above stocks can be found at the website of the Bloomington Drosophila Stock Center (www.flybase.org).

All *Drosophila* stocks were maintained on corn meal/ yeast extract/raisins at a temperature of 25 °C.

### Deficiency mapping and complementation testing

Each stock bearing an ethyl metanesulphonate (EMS)-induced lethal *dfmr*1*-*modifier mutation [44] was crossed to several duplication stocks from the X chromosome collection of Bloomington Drosophila Stock Center. If a particular duplication ‘rescued’ this lethal modifier mutation, its cytological position was defined within the duplicated region. Each cross was performed in duplicate and over 150 flies were scored.

Standard complementation tests were performed with each mapped modifier mutation in order to identify the correct candidate allele from the Bloomington Stock Center, residing in the same cytological region. These tests were based on lethality so that two allelic lethal mutations combined by crossing in the same genotype killed the F_1_ progeny.

### Dose-sensitive experiments

We performed dose-sensitive experiments in order to study the genetic interactions of the modifier mutations *peb ^hnt-E^* ^8^, *rok* ^1^, *sgg^G^* ^0263^ and *ras^G^* ^0380*b*^ with the gene *dfmr*1 in the adult brain. We designed stocks that were heterozygous for both *dfmr*1 and each of the modifier mutations and expressed GFP in the pdf neurons:

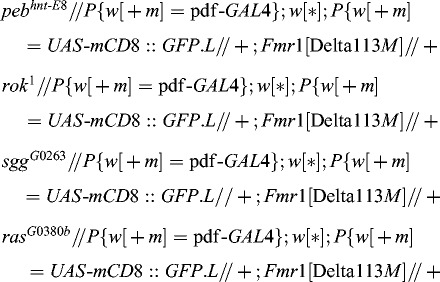



### Immunohistochemistry and confocal microscopy

Immunocytochemisty was performed on brain whole mounts as described earlier.[46] Adult brains from female flies were dissected in ice-cold phosphate buffered saline (1x PBS, pH 7.2), fixed in 4% freshly prepared paraformaldehyde for 30 min at room temperature and washed three times in 1x PBS (5 min each). The brains were mounted in 9:1 glycerol and 1x PBS. Microscope analysis was performed under laser-scanning confocal microscope Leica TSC SPE Microystem (ReProForce 2009-12-01, FP7-REGPOT) with photomultiplier and LAS Leica image software. Brain morphology was assessed by scanning with an HC PL APO40 x objective and by performing optical sections (*z* = 1.0 μm). For each experiment 8–14 half-brains were observed. Images were processed with Adobe Photoshop CS.

### Neuron morphology analysis

To obtain information on the fluorescence intensity of the small LNv (s-LNv's)-dorsal projections, we used the software Image J available online (http://imagej.en.softonic.com). Regions of interest (ROIs) were drawn around the axonal projections of the s-LNv's, starting from the beginning of each axon and ending in the point of its bifurcation; and the following values were calculated: AREA, INTEGRATED DENSITY and MEAN. The same procedure was repeated for a background area for each image. Values of corrected total fluorescence (CTF) were calculated and averaged for each genotype. Different genotypes were compared by using the two-tailed *t*-test (GraphPadIn Stat 3.01, see below).

To obtain information on the synaptic areas of the s-LNv's, we used the approach described before.[35] Two measurements were used for the analysis of these areas: target-area length and target area-width. All values were corrected against ½ of the posterior optic tract (POT) length. The area coverage was calculated by multiplying the uncorrected target-area length by the uncorrected target-area width then corrected against the square of the POT length.

All experiments with the suppressor mutations were compared to the wild type. All experiments with the double heterozygotes were compared to the corresponding single heterozygote and to *dfmr*1 (null) – heterozygote. POT splitting phenotypes were also analysed. If a POT displayed separation over a quarter of its length, it was regarded as ‘split’.

### Western blot analysis

Protein lysates were prepared from pharate adults or from embryos. An equal number of fly heads/embryos was isolated for each experiment, homogenized in radioimmunoprecipitation assay (RIPA) buffer (0.1% Triton X-100, 10% glycerol, 0.04% Na-deoxycholate, 50 mmol/L 4-(2-hydroxyethyl)piperazine-1-ethanesulfonic acid (HEPES) at pH 7.5, 2 mmol/L ethylenediaminetetraacetic acid (EDTA) and 100 mmol/L NaCl). Phosphatase Inhibitor Cocktail (10x, Sigma), 1 mmol/L NaVO_4_ and 100 mmol/L NaF were also added. Total protein concentration was measured by the Epoch Micro-Volume Spectrophotometer System (BioTec). The following antibodies were used: monoclonal anti-dFMR1 6A15 (1:1000, Sigma), monoclonal anti-α-tubulin (1:5000, B512, Sigma), monoclonal anti-Hindsight 4F3 (1:500, Creative Diagnostics), monoclonal anti-IMPDH2 (1:250, Millipore), polyclonal anti-Rock1 H-85 (1:250, Santa Cruz Biotechnology), monoclonal anti-GSK3 4G-1E (1:250, Millipore) and polyclonal p-GSK -3β - Ser 9 (1:250, Santa Cruz Biotechnology). We used the following secondary antibodies: goat anti-mouse IgG-HRP and goat anti-rabbit IgG-HRP (1:3000, Santa Cruz Biotechnology). Proteins were detected by the enhanced chemiiluminescence (ECL) method (Western Blotting Luminol Reagent Kit, Santa Cruz Biotechnology), normalized to α-tubulin and quantified by the software Image J 1.47v.

### Statistical analysis

Statistical analyses for the different genotypes were performed by using the two-tailed *t*-test with the software GraphPad InStat 3.01. All significant levels of differences between the genotypes were represented as *p* < 0.05 (*), *p* < 0.01 (**) and *p* < 0.001 (***). Error bars represent the standard error of the mean (SEM) for the Western blot analyses and standard deviation (SD) for the analyses of neuronal morphology.

## Results and discussion

### Genetic screen for modifiers which interact with *dfmr*1 in the wing tissue of adult *Drosophila*

**Figure 1.**
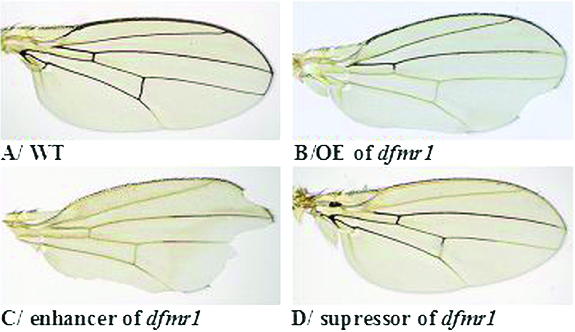
Wing phenotypes of flies with different genotypes: wild-type wing (A); overexpression of *dfmr*1 causes ‘notched’ wing phenotype (control) (B); enhancer wing phenotype (C); suppressor wing phenotype (D); overexpression of *rok* (E); overexpression of *sgg* (F).

In previous experiments we carried out a genetic screen for EMS-induced X-chromosomal modifier mutations of *dfmr*1. We isolated dominant enhancers and suppressors of the mutant wing phenotype induced by overexpression of *dfmr*1 in the wing margins of adult flies – ‘notched wings’ with missing wing hairs (genotype *GAL*4*-vg.M//+*; *UAS-Fmr*1*.Z//+*, Figure 1(B)). This phenotype was consistent with an earlier report.[26] The isolated mutations dominantly modified this control phenotype. The enhancers exhibited more prominent defective wing phenotypes and the suppressors had wild-type wings (Figure 1(C) and 1(D)). For further analysis we took only those suppressors and enhancers that had a recessive lethal effect on the viability of flies, e.g. loss-of-function mutations, and mapped them by means of the classical recombination analysis. We assumed that such mutations arose in genes interacting with *dfmr*1 and functioning in a common biological pathway. These initial results of our genetic screen were reported recently.[44]

In the present study we determined the exact location of most of these modifying mutations in the corresponding regions of the *Drosophila* cytological map. We used a set of overlapping duplications in the same regions of the X chromosome where the mutations were mapped previously by analysis of the meiotic crossing-over.[44] The duplications that ‘rescued’ the male individuals bearing a lethal mutation determined its position within individual discs from the X chromosomal polytene map.

We selected a group of candidate genes which were located in the same cytological regions where the *dfmr*1-modifying genes were mapped and which had suitable stocks with lethal alleles available through the Drosophila Stock Center. By means of the standard complementation test we checked which of the selected lethal alleles of the candidate genes for each modifying mutation did not complement this mutation.

That is how we identified four alleles: *peb^hnt-E^* ^8^ (аmorphic allele), *rok* ^1^ (ЕМS-induced allele), *sgg^G^* ^0263^ (P-element induced allele) and *ras^G^* ^0380*b*^ (P-element induced allele), belonging to four different genes, respectively: *pebbled/hindsight* (*peb/hnt*), *rok* (*rok*), *shaggy* (*sgg*) and *raspberry* (*ras*). Each of these lethal alleles showed lack of complementation when combined in the same genotype with the corresponding modifier suppressor mutation of the *dfmr*1 overexpression phenotype. As a last step in the identification of these alleles, we investigated their genetic interactions with *dfmr*1 in the adult wings. As expected, all four alleles suppressed the mutant wing phenotype of *dfmr*1.

### The genes *pebbled*, *rok*, *shaggy* and *raspberry* are dominant suppressors of the ‘notched’ wings induced by overexperession of *dfmr*1

The finding of genetic interactions between *dfmr*1 and specific alleles of *pebbled*, *rok*, *shaggy* and *raspberry* posed the question whether these interactions refer to other alleles of the same genes. As *peb^hnt-E^* ^8^, *rok* ^1^, *sgg^G^* ^0263^ and *ras^G^* ^0380*b*^ are lethal alleles, we also checked for genetic interactions of *dfmr*1 with other lethal alleles of the genes *shaggy*, *rok* and *raspberry*, for which stocks were available from the Flybase in Bloomington (*rok* ^2^, *sgg^G^* ^0335^, *sgg^G^* ^0183^, *ras^G^* ^035^, *ras^G^* ^048^). For the *pebbled/hindsight* gene, there are at least 14 lethal alleles, but there are no commercially available stocks.

For all alleles analysed, we observed a dominant suppressor phenotype: ‘correction’ of the ‘notched’ wing phenotype, similar to that described for the alleles in the previous part of our results (Figure 1(D)). These results clearly indicate that the interactions with *dfmr*1 refer to the genes, not to their specific alleles, suggesting that these interactions reflect a common biological function. Based on this, we considered the loci *rok*, *sgg*, *ras* and *peb* as functional partners of *dfmr*1 in the process of *Drosophila* wing development.

We undertook our research with the aim to isolate new genes whose protein products are functional partners of *Drosophila* FMRP. We focused on the X-chromosomal part of the genome because a genetic screen for autosomal genetic interactors of dRMRP has already been conducted.[39] In our work, we used several assays to look for the genetic interactions between *dfmr*1 and EMS-induced X-chromosomal mutations.[44] In the first assay, we identified four genes with mutations suppressing the ‘notched’ wing phenotype. Two of these genes encode proteins with kinase activity: the Rho kinase Rok and the Glycogen synthase kinase Shaggy. The other two suppressors were identified as the transcription factor Peb/HNT and the inosine-5′-monophosphate (IMP) dehydrogenase Ras.

Being a nuclear zinc-finger protein, Peb is known to regulate different morphogenetic processes in *Drosophila* development: the germband retraction during the embryonic stage,[47] the midgut and tracheal development,[48] the rhabdomere formation and the retinal epithelium integrity.[49] Here, to the best of our knowledge, we provide the first data that Peb functions together with dFMRP in a common pathway during wing development. It is known that an important role in the development of the wing margins is played by the Wnt/Wg-pathway.[50–53] We speculate that dFMRP and Peb, which work together in wing development, may execute their function through the Wnt/Wg-pathway. The Wg-canonical pathway, when activated, causes accumulation of Armadillo (*Drosophila* homologue of mammalian β catenin), which translocates into the nucleus and together with unknown transcriptional factors co-activates specific gene expression.[54] As Peb is a transcription factor and as dFMRP is involved in chromatin-mediated regulation of gene expression,[55,56] we are tempted to speculate that dFMRP and Peb may be part of this transcriptional machinery. Alternatively, Peb and dFMRP may act on wing development through another non-canonical Wg-pathway or through a different signalling pathway.

The identification of Rok as an interactor of dFMRP is not surprising, as the Rho kinases are known to be involved in the development of epithelial tissues, including the wing tissue. They all require highly regulated cell shape change, migration and rearrangements accomplished by dynamic changes of the actin cytoskeleton.[57] Our genetic data show that dFMRP and Rok function together in wing development, whereby dFMRP is upstream to Rok.

We also found that the Glycogen synthase kinase GSK3 (GSK3β)/Shaggy is another interactor of dFMRP in the wing tissue of *Drosophila*. Its identification is a proof for the specificity of our assay, as GSK3 has been reported to interact with mouse FMRP in adult neurogenesis. Other studies showed that GSK3 mRNA and protein levels were negatively regulated by FMRP.[58] Our genetic experiments demonstrated that Shaggy and dFMRP are functional partners in wing development and dFMRP acts, most probably, upstream in a pathway. *Drosophila* GSK3 (Shaggy) is considered an antagonist of the Wg-signal transduction in the Wnt/Wg pathway. This pathway plays an important role in wing patterning and development.[59,60]

The IMP-dehydrogenase (inosine-5′-monophosphate dehydrogenase, IMPDH) Ras, converts inosine monophosphate to xanthosine monophosphate and is responsible for the production of guanine nucleotides in the *de novo* GTP biosynthesis.[61] In *Drosophila* this protein has been shown to directly modify the Rho signalling and the actin cytoskeleton,[62] which may explain its involvement in the development of the wings.

### The modifier mutations affect dominantly axon and synaptic morphology of brain neurons

In order to elucidate the functions of the identified dominant modifier mutations in a more relevant physiological context, we analysed their role in the adult nervous system. We chose to investigate the axonal and synaptic morphology of a group of well-studied brain neurons: the LNv's expressing the neuropeptide PDF.[46,63,64]

In our experiments we used the GAL4/UASsystem and overexpressed in the adult LNv's the mCD8::GFP transgene, which labelled the neuronal membranes and the neuronal projections (see the ‘Materials and methods’ section). These pdf neurons consist of two subgroups: small and large ventral and lateral neurons – s-LNv's and l-LNv's. The s-LNv's send their axonal projections and arborize in the dorsal protocerebrum, while the l-LNv's send projections to the contralateral optic medulla trough the POT.[65]

**Figure 2.**
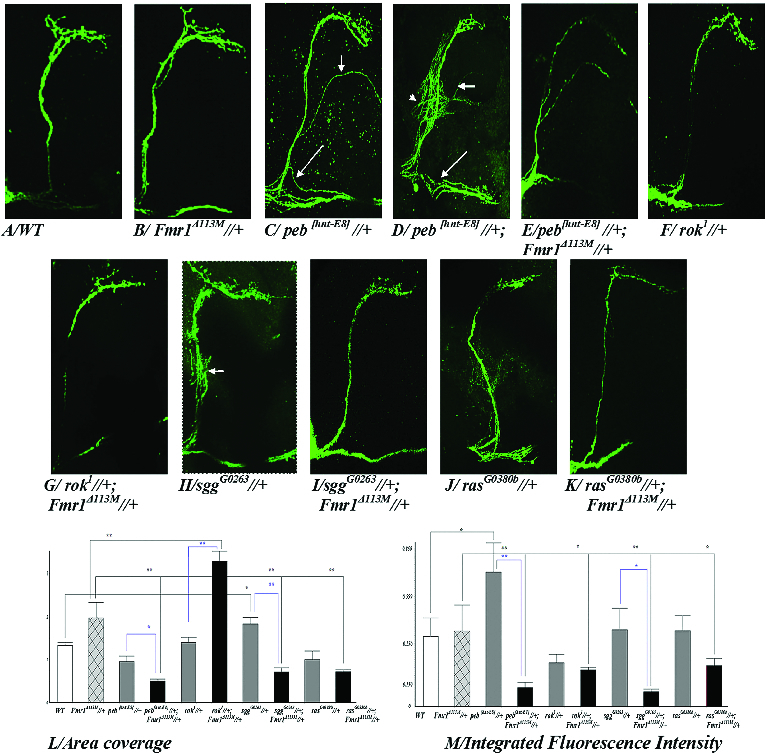
Confocal images of LNv's from adult half-brains with different genotypes: wild type (A); heterozygotes for the *dfmr*1-null mutation (B); heterozygotes *peb* ^[*hnt-E*8]^ *//+* (C); heterozygotes *peb* ^[*hnt-E*8]^ *//+* (D); double heterozygotes *peb* ^[*hnt-E*8]^//+; *Fmr*1^Δ113*M*^//+ (E); heterozygotes *rok* ^1^ *//+* (F); double heterozygotes *rok* ^1^ *//+*; *Fmr*1^Δ113*M*^//+ (G); heterozygotes *sgg^G^* ^0263^ *//+* (H); double heterozygotes *sgg^G^* ^0263^//+; *Fmr*1^Δ113*M*^//+ (I); heterozygotes *ras^G^* ^0380^ *^b^//+* (J); double heterozygotes *ras^G^* ^0380^ *^b^//+*; *Fmr*1^Δ113*M*^//+ (K). The target-area coverage for each genotype (L); Mean fluorescence intensity (M). All brains are shown at 40× magnification. Error bars indicate SD.

We first looked for the effect of the identified modifier mutations themselves, which are allelic to: *peb^hnt-E^* ^8^, *ras^G^* ^0380^ *^b^*, *shaggy^G^* ^0263^ and *rok* ^1^ (designated further with the symbols of these alleles), on the architecture of the LNv's and their synapses. A representative picture of adult half-brains from flies, heterozygous for each of these mutations is shown in Figure 2.

We quantified the defects in the dorsal arborizations of the s-LNv's and in the morphology of the POTs, generated by the axonal commissures of the l-LNv's. To assess the effects of the suppressor mutations identified in this study on the axonal growth and synapse architecture, we measured the fluorescence intensity of the s-LNv axons (mean fluorescence intensity) and the synaptic area (target-area coverage) that all modifier mutations affected the axonal and synaptic morphology dominantly, demonstrating severe aberrations in their growth, pathfinging and branching.

Adult brains with a *peb^hnt-E^* ^8^-heterozygous genotype showed ectopic dorsal branching (Figure 2(C) and (D), Table 1) and strongly defasciculated axonal bundles of the s-LNv's (Figure 2(D), Table 1). The mean fluorescence intensity of the s-LNv axons in these heterozygotes was significantly increased compared to that in wild-type flies (Figure 2(M)). Most probably this might be due to axon–axon repulsion within the axon bundle.

**Table 1.** Ectopic branching and defasciculated axonal bundles of *Drosophila* LNv's.

	*WT*	*peb*^[^*^hnt-E^*^8]^//+	*rok*^1^//+	*sgg^G^*^0263^//+	*ras^G^*^0380*b*^ //+
Ectopic axonal branching (per cent of total half-brains)	0%	60%	0%	13%	0%
	0 (6)	6 (10)	0 (10)	2 (15)	0 (10)
	*n* = 6	*n* = 10	*n* = 10	*n* = 15	*n* = 10
Defasciculated s-LNv-axon bundle (per cent of total half-brains)	0%	60%	40%	33%	40%
	0 (6)	6 (10)	4 (10)	6 (15)	4 (10)
	*n* = 6	*n* = 10	*n* = 10	*n* = 15	*n* = 10
Defasiculated bundle of POT (per cent of total half-brains)	0%	50%	10%	40%	80%
	0 (6)	5 (10)	1 (10)	6 (15)	8 (10)
	*n* = 6	*n* = 10	*n* = 10	*n* = 15	*n* = 10

The synaptic area of the s-LNv's also displayed abnormal architecture. Its size (expressed as target-area coverage) was decreased in comparison to the wild type (Figure 2(C), 2(D) and 2(L)). POTs often displayed abnormal phenotypes due to defasciculation of their commissures (Figure 2(C) and 2(D), Table 1). Fifty per cent of the half-brains analysed in our study had this mutant phenotype.

In earlier works Peb has been reported to play an important role in eye development.[47,49] Later, Oliva and Seirralta observed that the protein is involved in neuronal morphogenesis.[66] They showed that both overexpression and underexpression of Peb affected the axon growth, pathfinding and axon–axon interaction of the photoreceptor neurons. The results from our experiments demonstrate that the correct amount of Peb is important for the axonal and synaptic morphology, which could reflect its role in axon growth, pathfinding and branching. They emphasize the role of Peb in the transcriptional control of these processes and in establishing the proper wiring in the brain.

We next examined the pdf neurons in *rok* ^1^-heterozygous adult brains. It can be seen from Figure 2(F) that the dorsal projection of s-LNv's looked thinner than that of the wild type (though their mean fluorescence intensity did not differ significantly from that of the wild-type s-LNv's). Many of them (50%) had defasciculations (Table 1). Their synapses also looked defective, even though without a change in their target-area coverage (Figure 2(L)). We only rarely observed defasciculations in the POTs (Table 1).

The serine-threonine kinase Rok (encoded by *rok*) is a downstream effector target of the GTPase Rho, and thus part of the Rho-signalling pathway.[67] This pathway has important roles in different aspects of neuronal development, including neurite outgrowth, extension and branching and pathfinding.[68]

Our results clearly demonstrated that, similarly to *peb*, the correct dose of the *rok* product is required for the normal axon growth and synapse formation of the brain neurons. In *Drosophila* Rok phosphorylates the myosin regulatory light chain (MRLC), which is encoded by the gene *spaghetti squashs* (*sqh*). This phosphorylation is a signal for actin reorganization.[69] It can be speculated that Rok mediates its function in axon or synapse growth and morphology of brain neurons via control of actin or microtubulae reorganization.

The structure of the LNv axons and their synapses in *sgg^G^* ^0335^ *-*heterogygous adult brains is shown in Figure 2(H). The bundle of the dorsal s-LNv-axon projections often looked less organized and defasciculated (Figure 2(H)). Their mean fluorescence intensity was not significantly higher than that of the wild type (Figure 2(M)). In half of the cases, we observed POT defasciculations (Table 1). The synaptic area of the s-LNv often displayed a defective, disorganized structure with ectopic branching, as compared to the wild type, which made it significantly larger than that of the wild type (Figure 2(L)).

Earlier works have shown that GSK3 negatively regulates axon formation in mammalian neuronal cultures.[70] In the peripheral nervous system this kinase is required presynaptically and controls the growth of the neuro-muscular junctions (NMJ) through phosphorylation of the microtubule-binding protein Futsch.[71]

Our results on the axon and synaptic morphology of the pdf neurons in brains heterozygous for *sgg^G^* ^0263^ demonstrate that Shaggy is most probably required for the correct morphology of axons and synapses in *Drosophila* brain. The pathway of Shaggy which is related to the control of these neuronal processes is so far unknown. Most probably, it targets the microtubule/actin cytoskeleton dynamics in the Wnt/Wg pathway. There is growing evidence that this pathway regulates different aspects of the nervous system development and the structure and function of the adult nervous system.[72,73]

We also studied the pdf neurons in *ras^G^* ^0482^-heterozygous adult brains. Their axonal morphology and synapses are shown in the representative picture in Figure 2(J). The dorsal axons of the s-LNv's looked strongly underbranched in most of the half-brains examined. These dorsal axons terminate their distal arborizations with a dramatically reduced and collapsed synaptic area (Figure 2(J) and 2(L)). The majority of l-LNv's (80%) showed a spit-POT phenotype.

The *ras^G^* ^0482^ allele is a P-element induced allele of the gene *raspberry* encoding the enzyme inosine monophosphate dehydrogenase (IMPDH), which is involved in *de novo* synthesis of guanine nucleotides. It has been reported that this *de novo* synthesis is required for the photoreceptor axon guidance and pathfinding in *Drosophila* visual system.[74] Our results extend the importance of inosine-5′-monophosphate dehydrogenase for the formation of neuronal circuits in the brain. We demonstrated that this enzyme's function is important for the axon morphology and the synaptic branching of the brain LNv's. No ectopic branching was found in the dorsal axons of s-LNv's (Table 1), nor a significant difference of the mean fluorescence intensity of these axons compared to the wild type (Figure 2(L)). However, we observed aberrant synaptic architecture in many of the half-brains analysed, even though the quantification of this result did not show a significant difference compared with the wild type.

So far it is not clear how IMPDH, which is a housekeeping component of every organism and controls the entry of purines into the pool of guanine nucleotides,[75] executes its neuronal function. One possibility is that *de novo* guanosine monophosphate (GMP) synthesis may be used for the activation of the Rho-signalling pathway and the subsequent actin reorganization.[74] Alternatively, the regulation of axon and synaptic development may involve a ‘moonlighting’ function of Ras: RNA-binding and RNA metabolism regulation, including translation, stability or splicing. A similar function in the translational regulation was reported for the mammalian IMPDH.[76] It can be hypothesized that Ras might bind an mRNA for a cytoskeletal component and might regulate its protein abundance. The importance of the purine metabolism for the nervous system in humans may be illustrated by some metabolic disorders which affect the nervous system and which are caused by mutations governing this metabolism.[77,78]

### The modifier mutations act together with *dfmr*1 in the axonal growth and synaptic architecture of adult brain neurons

To explore the genetic interactions between *dfmr*1 and the modifier mutations in the fly nervous system, we investigated the phenotypes of the adult pdf neurons from genotypes with half-reduced gene doses of *dfmr*1 and the isolated modifier mutation (genotype: suppressor mutation//+; *dfmr*1-null//+). We analysed the growth of the dorsal s-LNv axons by examining their mean fluorescence intensities and assessed the overall architecture of their synapses by determining the coverage of target areas. The results of our observations are presented in Figure 2(E), 2(G), 2(I) and 2(K)–(M)). Confocal images from the double heterozygous adult flies are presented next to those from the single heterozygote for the corresponding modifier mutation.

We carried out these dose-sensitive experiments, expecting that, if the two genes in the double heterozygote performed a common biological function, due to the misbalance of their common pathway, the double heterozygote would have a ‘worse’ axonal and synapse phenotype than the phenotype of the single heterozygote for the modifier mutation (Flies heterozygous for the *dfmr*1-null-mutation, e. g. *Fmr*1^Δ113*.M*^//+, had the same pattern of axon and synapse growth of the pdf neurons as that of wild type – Figure 2(B), 2(L) and 2(M)); Table 1). Our observations confirmed these predictions and demonstrated that in all cases the double heterozygotes had considerably ‘worse’ s-LNv-axonal growth phenotypes. It can be seen from Figure 2(E), 2(G), 2(I) and 2(K) that the dorsal axon bundles of the s-LNv's from double heterozygous adult brains looked thinner and more undergrown in comparison to the heterozygotes for any single modifier mutations (*peb^hnt-E^* ^8^, *rok* ^1^, *sgg^G^* ^0263^ and *ras^G^* ^0380*b*^). The mean fluorescence intensities measured for these axons showed significantly much lower values as compared to the heterozygote *Fmr*1^Δ113*.M*^//+. Besides, they had lower values when compared to the corresponding single heterozygotes, especially for *peb^hnt-E^* ^8^ and *sggG*0263 (*p* < 0.05). For *rok* ^1^ and *ras^G^* ^0380^ these differences were less prominent and were statistically non-significant (due to the relatively low number of the half-brains analysed) (Figure 2(M)).

The same tendency of ‘worsening’ was also valid for the synaptic architecture of the double heterozygotes, where their defects were even more dramatic. Synaptic areas were strongly reduced for genotypes with one copy of the normal *dfmr*1 gene and one copy of either of the modifier mutations: *peb^hnt-E^* ^8^, *sgg^G^* ^0263^ and *ras^G^* ^0380*b*^ (Figure 2(G), 2(I), 2(K) and 2(L)), or they were even almost completely absent (Figure 2(E)).

The only modifier mutation which had a significantly increased coverage of the target area was *rok* ^1^ (Figure 2(L)), regardless of the strongly disrupted synapses in all the half-brains examined. This observation may be explained by the way the value of the target-area coverage was obtained (in arbitrary units). It is a product of the target area width and the target-area length. The synaptic areas of the double heterozygotes with *rok* ^1^ and *dfmr*1 were much longer, though thinner, than those of the double heterozygotes with the other three modifier mutations (values for target-area width and target-area length are not presented in Figure 2(L)). Thus, the extensive coverage of the target area may be explained by an increased extension of the terminal axonal projections. As neither of the single heterozygotes (*dfmr*1*//+* or *rok* ^1^//+) alone showed overextension phenotypes, we could explain this observation as the evidence for genetic interaction between *dfmr*1 and *rok* ^1^ and for their common function in the process of axon growth and extension.

Altogether, our results demonstrated that the modifier mutations *peb^hnt-E^* ^8^, *rok* ^1^, *sgg^G^* ^0263^ and *ras^G^* ^0380*b*^ isolated as suppressors of the *dfmr*1-overexpression wing phenotype interact genetically with *dfmr*1 in the context of the nervous system. The proteins they encode, Peb/Hnt, Rok, Shaggy and Ras, produce phenotypes previously described for mutants lacking dFMRP. Numerous studies have shown that dFRMP was required for normal axonal and synaptic growth, extension, guidance and branching in both the central and the peripheral nervous system of *Drosophila*.[79–84] Based on our observations, we suggest that Peb/Hnt, Rok, Shaggy and Ras are functional partners of dFMRP in the axonal and synaptic growth and synapse formation of adult brain neurons.

**Figure 3.**
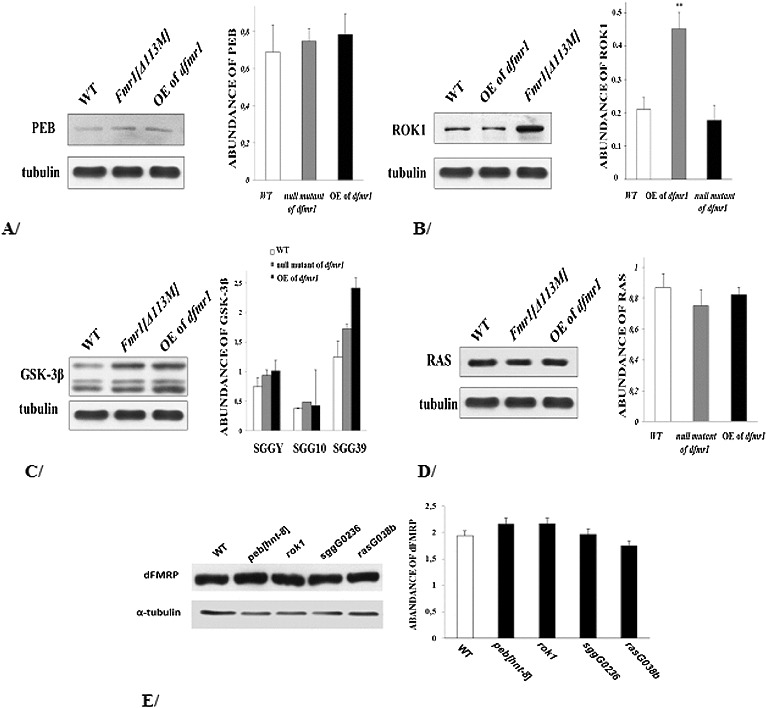
Western blot analysis. Expression levels in wild-type, *dfmr*1-null mutants and in the case of *dfmr*1 overexpression in the brains of: Peb/HNT – about 209 kDa (A); Rok – about 160 kDa (B); Shaggy/GSK3β (C) for each isoform: SGGY (about 56 kDa), SGG10 (about 58 kDa) and SGG39 (about 68 kDa); Ras – about 60 kDa (D). Expression levels of dFMRP (about 85 kDa) in embryos hemizygous for *peb* ^[*hnt-E*8]^, *rok* ^1^, *sgg^G^* ^0263^ or *ras^G^* ^0380*b*^ (E) (*p* > 0.05; *n* = 3). Quantification of each protein expression from Western blot analysis is shown on the right side. Error bars indicate SD.

To better understand how these proteins interact with dFMRP, we conducted biochemical assays. We first performed Western blot analysis of embryos mutant for each of the modifier mutations and looked for their dFMRP abundance. We found no significant difference in the level of expression of this protein in either of the mutants as compared to the wild type. A representative Western blot is shown in Figure 3(E) (*p* > 0.05, *n* = 3). The quantification of dFMRP expression from Western blot analysis is shown on the right side of the blot. The band size is about 85 kDa. Based on these results we concluded that neither of the modifier genes encoding Peb/Hnt, Rok, Shaggy or Ras controlled the expression of dFMRP.

We also performed Western blot analysis of brains from pharate adult flies, where dFMRP expression was known to be high,[83] and looked for the expression levels of the proteins Peb, Rok, Shaggy and Ras in *dfmr*1-null genotypes or in such with pan-neuronal expression of dFMRP (*GAL*4*-elav.L//UAS-Fmr.Z*). As the antibodies we used in this analysis were not created against the *Drosophila* proteins Rok, Shaggy/GSK-3β and Ras but their mammalian homologues, we first carried out an experiment to confirm that the antibodies did recognize these proteins as well (data not shown). Representative Western blot images for the levels of Peb, Rok, Shaggy and Ras are presented in Figure 3(A)–(D). The quantification of each protein expression from the Western blot analysis is shown on the right side of the blot.

The level of Rok (molecular weight of about 160 kDa) was significantly increased to 2.1 times of the wild-type level (**p < 0.01, *n* = 4) in the *dfmr*1-null mutants. In flies overexpresing *dfmr*1 the level of the protein was not different from that of the wild type (Figure 3(B)). From these results we infer that dFMRP negatively regulates the protein level of Rok. It might be suggested that dFMRP, which is a well-known translation regulator, negatively controls the abundance of the Rho kinase Rok affecting the phosphorylation of its downstream targets, actin cytoskeleton remodelling and axonal and synaptic features. The expression level of this key protein is expected to be tightly regulated and dFMRP might be an important contributor in this process. Being a key molecular switch to cytoskeleton events, Rho kinase is considered an important pharmaceutical target in some neurological diseases, like stroke, inflammatory and demyelinating diseases, where its inhibition prevents neuronal degeneration.[85] Our results suggest that Rho kinase might be a good candidate for a therapeutic target in FraX syndrome, similarly to other neurological disorders.

The abundance of Peb (band size of about 209 kDa) did not show any significant differences in either of the mutant genotypes analysed as compared to the wild type (Figure 3(A); *p* > 0.05, *n* = 3). Ras expression levels (about 60 kDa) also displayed no difference between the *dfmr*1-null mutants, the mutants overexpresing *dfmr*1 and the wild type (Figure 3(D); *p* > 0.05, *n* = 4).

To assess the abundance of the protein Shaggy, we used an anti-GSK3 mouse monoclonal antibody which recognizes a synthetic peptide corresponding to the catalytic domain of the *Drosophila* Shaggy enzyme (anti-GSK3, clone 4G-1E, Millipore). This antibody detected three different isoforms in the pharate adult heads with the following sizes: 56 kDa, 58 kDa and 68 kDa, which have also been found in larval, pupal and adult protein lysates by Ruel et al.[86] These isoforms were termed, correspondingly: SGGY, SGG10 and SGG39. In two Western blots from pharate adult heads we did not observe any difference in the protein abundance of the three Shaggy isoforms between the *dfmr*1-null mutants, the mutants overexpresing *dfmr*1 and the wild type (Figure 3(C)).

This last result is in a partial accord with the data of Min et al.,[87] who also found that total levels of GSK3 (α- and β-proteins) were equivalent in wild-type and in mutant FraX knockout mice. These authors reported though, that the active, unphosporylated form of GSK3 was elevated in specific regions of FraX mouse brain. On the other hand, in the FraX mouse model other authors found that mutant knock-out mice lacking FMRP had an elevated level of the total protein, suggesting a negative control of this enzyme by FMRP.[88] Furthermore, there is important evidence that inhibitors of GSK3 activity, including lithium (a mood stabilizer), contributed to the treatment of many of the FraX phenotypes in mice,[89] and this suggested that hyperactive GSK3 in such mice was part of the pathological phenotypes. GSK3 is now considered a promising target for the therapeutic treatment of many neurological diseases.

*Drosophila* GSK3β is known to be a constitutively active enzyme, or to be inactivated upon certain cues via serine phosphorylation (Ser-9).[90] To confirm the importance of the inactivation of GSK3β in the *Drosophila* brain and a possible requirement for dFMRP in this process, additional biochemical and genetic experiments are needed.

Even though our genetic screen for *dfmr*1-modifiers is not exhaustive, it is interesting to note that the four genes found in this screen encode proteins which seem to be functionally linked to the Rho-pathway and to cytoskeletal events through the non-canonical Wnt-pathway. Our findings underline the importance of the Rho- and the Wnt-signalling pathways for the pathophysiology of FraX syndrome.

## Conclusions

To summarize, our studies revealed four X-chromosomal genes encoding the proteins Peb, Rok, Shaggy and Ras, which are functional interactors of dFMRP. To the best of our knowledge, this is the first report showing a functional link between *dfmr*1 and these genes previously unrelated to it in the *Drosophila* model of FraX. Our findings may have important relevance to understanding the genetic basis of some neuro-developmental processeses and performing treatment of human pathologies, including FraX syndrome.
